# Key patterning genes contribute to leg elongation in water striders

**DOI:** 10.1186/s13227-015-0015-5

**Published:** 2015-04-28

**Authors:** Peter Nagui Refki, Abderrahman Khila

**Affiliations:** Institut de Génomique Fonctionnelle de Lyon, CNRS-UMR5242, Ecole Normale Supérieure de Lyon, Université de Lyon, Université Claude Bernard Lyon 1, 46 Allée d’Italie, 69364 Lyon, Cedex 07 France

**Keywords:** Water striders, Growth, Leg length, Developmental genes, Pattern formation

## Abstract

**Background:**

How adaptive phenotypes are shaped by the action of key developmental genes during ontogeny remains poorly understood. Water striders, a group of hemipteran insects, present a unique example of adaptation to life on the fluid water surface substrate. The group has undergone a set of leg modifications allowing them to efficiently move on the water surface and hence invade a variety of niches from ponds to open oceans. The elongated legs of water striders play a key role in generating efficient movement on the fluid by acting as propelling oars.

**Results:**

To determine the developmental mechanisms underlying leg elongation, we examined the function of the key developmental genes *decapentaplegic* (*dpp*), *wingless* (*wg*), *epidermal growth factor recepto*r (*egfr*), and *hedgehog* (*hh*) during embryonic development in the water strider *Limnoporus dissortis*. By analyzing expression patterns and RNAi knockdown phenotypes, we uncover the role of these genes in leg growth and patterning during embryogenesis. Our results indicate that *wg* and *egfr* contribute to the elongation of all the three segments of all thoracic legs, whereas *hh* specifies distal leg segments.

**Conclusions:**

Together, our results suggest that key patterning genes contribute to the dramatic elongation of thoracic appendages in water striders.

## Background

**Figure 1:**
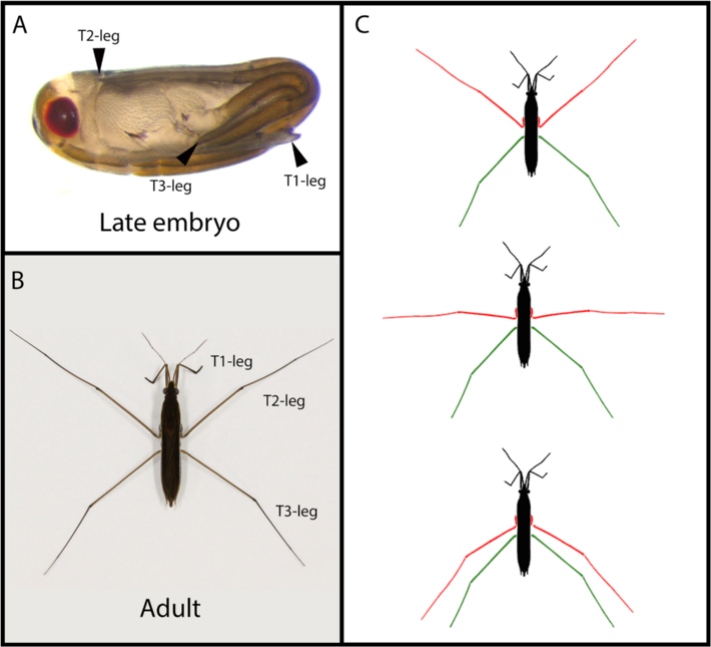
Leg ground plan is associated to the derived mode of locomotion of water striders. Leg allometry is established during embryogenesis in *L. dissortis*. **(A)** A late embryo showing T1 legs extending ventrally from the anterior to the posterior side, T2 legs folding dorsally reaching the head, and T3 legs extending laterally from side to side. **(B)** An adult *L. dissortis* individual showing the reversal in leg ground plan where T2 leg is longer than T3 leg, which in turn is longer than T1 leg. **(C)** This characteristic leg ground plan is associated to the mode of locomotion by rowing.

Adaptations at the organismal level represent a window to understand the interplay between selection and developmental genetic pathways in shaping phenotypic evolution [1]. However, the role of key patterning molecules in shaping adaptive morphologies during development is poorly understood. Water striders belong to a monophyletic group of semiaquatic heteropteran insects, also known as semiaquatic bugs, which represent a remarkable example of adaptation to water surface life, enabling them to invade various aquatic niches including open oceans [2]. Semiaquatic bugs follow a hemimetabolous mode of development where embryos hatch in a nymphal form which will undergo five successive molts to reach the mature adult stage [2]. This direct mode of development is associated with the completion of appendage specification and the scaling of their allometry by the end of embryogenesis (Figure 1A). In water striders, the length of all three appendages relative to body length has greatly increased compared to their terrestrial close relatives. In addition, T1 legs are shorter than T2 legs, which in turn are longer than T3 legs (Figure 1B) [2,3]. This derived ground plan is associated with a novel mode of locomotion of water striders where they use their T2 legs in simultaneous sculling motion to propel themselves while their T3 legs act primarily for orienting the animal (Figure 1C) [2]. This adapted mode of locomotion allows water striders to efficiently move on the fluid water-air interface.

The fine-tuning of leg allometry in water striders is largely driven by changes in the expression and function of the hox gene *Ultrabithorax* (*Ubx*) [3-5]. In many water striders including *Limnoporus dissortis*, Ubx is expressed in both mid- and rear legs and functions to elongate the mid-legs but to shorten the rear legs [3-5]. These opposing functions of Ubx are achieved through the emergence of sensitivity of leg tissues to differences in Ubx dose, thereby establishing the relative differences in length between T2 and T3 legs [5]. This novel ground plan of leg length is in turn associated with the evolution of water surface rowing as an efficient mode of surface locomotion. We have recently shown that Ubx exerts its role in modulating leg length by affecting the levels, but not the spatial pattern, of expression of a set of key developmental genes [5]. However, the role of these key developmental genes in scaling leg allometry in water striders remains to be tested. In addition, developmental genes such as *decapentaplegic* (*dpp*), *wingless* (*wg*), *epidermal growth factor receptor* (*egfr*), and *hedgehog* (*hh*) are known to play a key role in the growth and patterning of fly imaginal discs which give rise to adult appendages [6-9]. Here, we aim to determine the contribution of the four signaling molecules Dpp, Wg, Egfr, and Hh to the adaptive scaling of leg allometry in the water strider *L. dissortis*. Based on the observation that *dpp*, *wg*, *egfr*, and *hh* genes are expressed in the legs of *Limnoporus* embryos, and that *Ubx* gene knockdown using ribonucleic acid interference (RNAi) affects their levels, but not spatial patterns of expression [5], we hypothesized that they might contribute to the overall dramatic growth of water striders’ legs. We therefore examined their function using parental RNA interference [3,5] to determine their role in regulating the growth of the mid- and rear legs in the embryos of water striders.

## Methods

### Animal collection and rearing

*L. dissortis* adults were collected from the Acadie River, Montréal, Québec, Canada. Animals were kept at room temperature in containers filled with tap water and were fed on crickets. A piece of floating Styrofoam was supplied to the females to lay eggs on. Animal experimentation and manipulation were performed following guidelines of the decree number 2013-118 of 1st February 2013, Art. R. 214-88 of the *Ministère de l’agriculture*, *de l’agroalimentaire et de la forêt* (the French ministry of agriculture, agrifood, and forestry).

### Gene cloning

*Limnoporus* total RNA was extracted from different embryonic and larval stages. First-strand cDNA synthesis was then performed (Invitrogen, Waltham, MA, USA) using the total RNA as a template. *dpp*, *wg*, *hh*, and *egfr* were cloned through PCR using primers as described in [5]. Accession numbers are as follows: dpp KF630593; wg KF630597; hh KF630595; egfr KF630594.

### Embryo collection and dissection

Embryos were collected, treated with 25% bleach, and then washed with PTW 0.05% (1 × PBS; 0.05% Tween-20). For picture taking, late embryos were dissected out of the chorion, and pictures were captured either on live embryos or on embryos fixed with 4% formaldehyde. For staining, embryos of various early stages were dissected out of the chorion, cleaned from yolk as much as possible, and kept briefly in PTW 0.05% on ice until fixation with the appropriate fixation method according to the type of subsequent staining.

### *In situ* hybridization

Dissected embryos were fixed in 200 μl 4% paraformaldehyde (PFA) + 20 μl dimethyl sulfoxide (DMSO) and 600 μl heptane for 20 min at room temperature, then washed several times in cold methanol. Embryos were then rehydrated in decreasing concentrations of methanol in PTW 0.05% and washed in PTW 0.05% and PBT 0.3% (1 × PBS; 0.3% Triton X-100) three times each. Embryos were washed twice with PBT 1%, then transferred to 1:1 PBT 1%/hybridization solution (50% formamide; 5% dextran sulfate; 100 μg/ml yeast tRNA; 1× salts). The composition for 100 ml of 10× salts is as follows: 17.5 g sodium chloride, 1.21 g tris-base, 0.71 g monosodium phosphate, 0.71 g sodium phosphate dibasic, 0.2 g Ficoll 400, 0.2 g polyvinylpyrrolidone (*PVP*), 10 ml of 0.5 M EDTA, and 0.2 g BSA (pH 6.8). Embryos were pre-hybridized for 1 h at 60°C, followed by addition of a Dig-labeled RNA probe overnight at 60°C. Embryos were then transferred gradually from hybridization solution to PBT 0.3% through consecutive washes with 3:1, 1:1, and 1:3 (pre-warmed hybridization solution: PBT 0.3% gradient). A blocking step was performed with PAT (1 × PBS; 1% Triton X-100; 1% BSA) at room temperature followed by incubation with anti-dig antibody coupled with alkaline phosphatase for 2 h at room temperature. Embryos were washed several times in PBT 0.3% then in PTW 0.05% before color reaction is conducted with NBT/BCIP in AP buffer (0.1 M Tris pH 9.5; 0.05 M MgCl_2_; 0.1 M NaCl; 0.1% Tween-20).

### Parental RNAi

**Figure 2:**
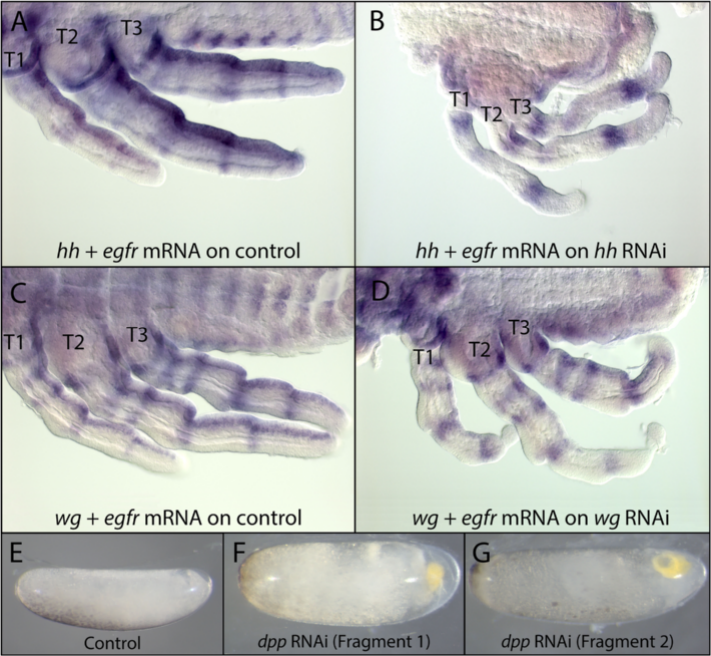
Efficiency and specificity of RNAi knockdown in *L. dissortis.* Embryos stained with a mix containing ten times diluted *egfr* probe as an internal control and *hh* probe **(A, B)** or *wg* probe **(C, D)**. (A) Normal embryo where the *hh + egfr* probe mix detected the expression of both *hh* and *egfr* mRNA. (B) *hh* RNAi embryo where the *hh + egfr* probe mix failed to detect *hh* but successfully detected *egfr* mRNA. **(C)** Normal embryo where the *wg + egfr* probe mix detected the expression of both *wg* and *egfr* mRNA. (D) wg RNAi embryo where the *wg + egfr* probe mix failed to detect *wg* but successfully detected *egfr* mRNA. **(E)** Early *Limnoporus* embryo developing normally. **(F, G)** dpp RNAi embryos showing the same phenotype. This phenotype was obtained using RNAi against two non-overlapping fragments of *dpp. dpp*: *decapentaplegic*; *egfr*: *epidermal growth factor recepto*r; *hh*: *hedgehog*; RNAi: gene knockdown using ribonucleic acid interference; T1, 2, 3: thoracic segments 1, 2, 3.

Double-stranded RNA synthesis was performed on a template flanked by T7 promoter sequences. For each gene, the template was generated by PCR using the following primers containing T7 promoter sequence which is depicted in lower case letters: *dpp*: forward M13-F primer positioned upstream of T7 promoter in pGEM-T vector (Promega, Madison, WI, USA) and reverse 5′-taatacgactcactatagggagaccacCTATGGCGGGCATGACATCCAAACT-3′; *wg*: forward 5′-taatacgactcactatagggagaccacCGGCATTCATMTATGCRATAACCAG-3′ and reverse 5′-taatacgactcactatagggagaccacTTGTATCCCTAGGCTCGGGTTGCGTT-3; *hh*: forward 5′-taatacgactcactatagggagaccacAAGACGAMGARGGCAGAGGWGCCGAT-3′ and reverse 5′-taatacgactcactatagggagaccacTARTWGACCCAATCGAAWCCGGCTTC-3; *egfr*: forward 5′-taatacgactcactatagggagaccacAAACGCACTTGCCAACGACTCAGAGTT-3′ and reverse 5′-taatacgactcactatagggagaccacAGTGGTAGGGTTGTATCGCTGCATTG-3. The two *dpp* fragments used in Figure 2 were generated using the following primers tagged with T7 promoter: *dpp* fragment1: forward 5′-taatacgactcactatagggagaccacTCTACTACAGCTACTCGGCATGCCCA-3′ and reverse 5′-taatacgactcactatagggagaccacCTTCTTGGGGTATTGAGACGTTAA-3′; and *dpp* fragment2: 5′-taatacgactcactatagggagaccacAAGAGCGGCTCAGAAGAAACACAGG-3′ and reverse 5′-taatacgactcactatagggagaccacTCTGCGTCGAATTTCTCCTACCTGCA-3′.

These fragments were used as a template for *in vitro* transcription using T7 RNA polymerase, generating both sense and anti-sense transcripts, at 37°C. Complementary single RNA strands are automatically annealed into double-stranded RNA (dsRNA) while the reaction progresses without any further treatment. dsRNA is then purified using Qiagen RNeasy purification kit and eluted in Spradling injection buffer [10]. *Limnoporus* adult females were anesthetized using carbon dioxide, immobilized on double sticky tape, and injected with 2 μl of dsRNA at 1 to 2 μg/μl concentration. Injected females were replaced on water tanks; embryos were collected on floating Styrofoam and allowed to develop at room temperature. Embryos were screened for phenotypes morphologically by examining leg sizes and other segment defects.

### Leg measurements

A sample of 10 embryos (*N* = 10) was used for each RNAi group. Two sets of measurements were recorded for each embryo: the egg length and the length of late embryo legs. After measuring embryo length, embryos were dissected, split longitudinally, and mounted on slides on Hoyer’s medium. Measurements for each leg segment of each pair of legs were recorded on a Zeiss microscope using the Axiovision or Zen software.

### Statistical analyses

Statistical significance in leg length between each RNAi group and the wild type was determined by performing an analysis of covariance (ANCOVA). Egg length was used as a covariate as a possible factor impacting leg length, and the mean value of each pair of leg segments was used as the dependent variable. In cases where some data sets do not meet the assumption of homogeneity of regression required to proceed with the ANCOVA test, an analysis of variance (ANOVA) was performed for the mean values of leg segments corrected for egg length by dividing the mean value of the leg segments with the corresponding egg length. Statistical analyses were performed using the SPSS software package (IBM Corporation, Armonk, NY, USA).

## Results

### Expression and function of *dpp* in *Limnoporus*

**Figure 3:**
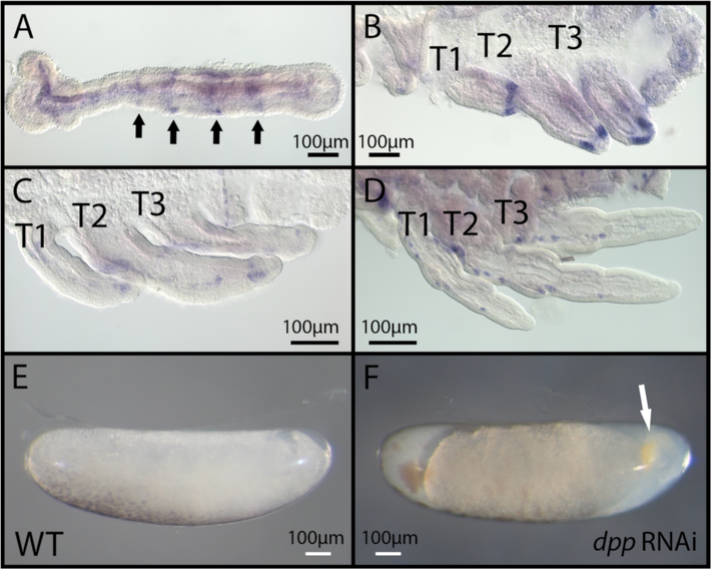
*dpp* expression and phenotypes generated by parental RNAi. **(A-D)** *In situ* hybridization revealing *dpp* mRNA expression in early embryos prior to limb bud formation (A), and embryos where the legs are extending (B-D). **(E)** Untreated embryo developing normally. **(F)** *dpp* RNAi resulted in halting embryonic development at an early stage. *dpp*: *decapentaplegic*; WT: wild type; T1, 2, 3: thoracic segments 1, 2, 3.

In early embryos and before the formation of limb buds, *dpp* mRNA is expressed in segmental stripes clearly visible in thoracic and head segments (arrows in Figure 3A). Later on, *dpp* expression forms a stripe at the distal appendages (Figure 3B). In later stages of embryogenesis, the distal stripe disappears, and *dpp* expression becomes fainter and distributed in patches along the proximo-distal axis of the legs (Figure 3C,D). *dpp* mRNA pattern of expression is consistent across all three legs, and no spatial variation or differential expression could be detected between legs [5]. Knockdown of *dpp* using RNAi resulted in severe defects such that embryogenesis was arrested at an early stage (compare panels E and F of Figure 3). This phenotype was also observed in *Oncopeltus* [11], suggesting a similar role for Dpp in hemimetabolous early development.

### Expression and function of *hh* in *Limnoporus* legs

**Figure 4:**
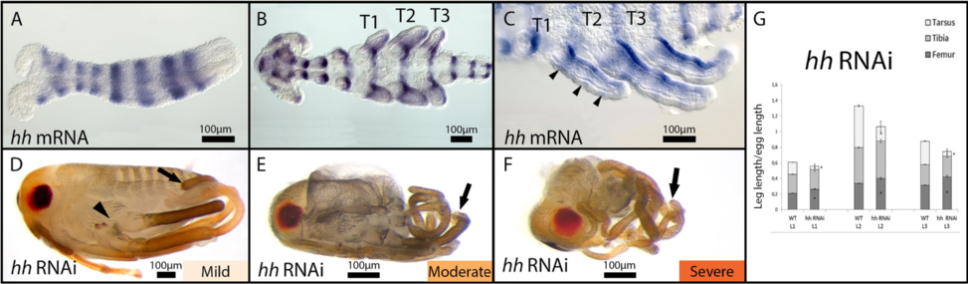
*hh* expression and function in embryonic leg development. **(A)** *hh* mRNA is expressed in early embryos in a segmental pattern along the anterior posterior axis. **(B)** The segmental expression persists and *hh* mRNA expands to the developing limb buds. **(C)** As the legs extend, *hh* mRNA becomes prominent in a posterior stripe in all the legs with additional lateral stripes (arrowheads). **(D)** Mild *hh* RNAi phenotype shows little segmentation defects such that the boundary between T2 and T3 segments appears to be fused (arrowhead). Mildly affected embryos show truncation of distal leg segments (arrow). **(E)** Moderate *hh* RNAi embryos showing patterning defects. **(F)** Segmentation defects aggravate in severe *hh* RNAi where only the head and the first thoracic segment could be distinguished, and all the legs are distally truncated. **(G)** Measurement of mild *hh* RNAi legs showing increased length of the femur in all three legs. Note that the tarsi and tibias in these embryos were partially or entirely missing. Leg measurements for T1 legs, T2 legs, and T3 legs are represented in the different bars; the three measured leg segments - the femur, the tibia, and the tarsus - are represented in the segments of each bar. *N* = 10; error bars represent ±1 standard error. ANOVA test performed using leg length divided by individual embryo length was used to correct for individual size variations. An asterisk indicates that the test was significant at (*P* ≤ 0.01). *hh*: *hedgehog*; RNAi: gene knockdown using ribonucleic acid interference; T1, 2, 3: thoracic segments 1, 2, 3; WT: wild type.

At early embryogenesis and before the formation of limb buds, *hh* is expressed in segmental stripes along the body axis of the embryo (Figure 4A). This segmental expression persists through later stages, after the formation of the appendages, where *hh* mRNA is detected at the posterior boundary of each segment along the germband (Figure 4B,C). In the extending embryonic legs, *hh* is strongly expressed along the posterior compartment and fades away towards the anterior. In addition to this gradient-like pattern, *hh* is expressed in relatively faint stripes along the proximo-distal axis of all the legs (arrowheads in Figure 4C). RNAi knockdown revealed the role of *hh* in regulating leg development of *Limnoporus* embryos (Figure 4D,E,F). Severe and moderate *hh* knockdown showed both segmentation defects and leg development defects (arrow in Figure 4D,E,F) indicating a conserved role of *hh* in insect segmentation [12]. The legs and antennae of severe *hh* RNAi phenotypes are thinner, indicating possible dorsal-ventral and/or anterior-posterior growth defects (Figure 4D,E,F). Mild *hh* phenotypes showed minor defects in patterning at the boundary between the second and third thoracic segments (arrowhead in Figure 4D) but showed clear defects in proximal-distal leg development. The proximal leg segments of mild *hh* phenotypes appeared normal, but distal segments showed a truncation where part or the entire tarsus was missing (arrow in Figure 4D,E,F). This indicates a role of *hh* in specifying distal segments of the legs during *Limnoporus* embryogenesis. Because RNAi against *hh* resulted in the truncation of distal legs, we did not consider distal appendages in our measurements of the role of *hh* in leg growth. However, we found that *hh* RNAi did not affect the length of the tibia but caused the elongation of the femur of all three thoracic appendages (*P* ≤ 0.01) without any patterning defects (Figure 4G; Table 1). This indicates that *hh* represses the growth of the femur in all legs.

**Table 1:** **Effect of RNAi on leg length as indicated by percentage increase (+) or decrease (−) in leg length**

	**T1 leg**			**T2 leg**			**T3 leg**		
**RNAi**	**Femur**	**Tibia**	**Tarsus**	**Femur**	**Tibia**	**Tarsus**	**Femur**	**Tibia**	**Tarsus**
*wg*	−15,59%	−19,26%	−18,13%	−31,35%	−32,09%	−40,35%	−24,62%	−24,18%	−36,35%
*hh*	+24,33%	+4,06%	Truncated	+18,71%	+4,09%	Truncated	+35,39%	−0,33%	Truncated
*egfr*	−14,65%	−14,90%	−35,15%	−36,75%	−31,19%	−43,15%	−30,55%	−23,82%	−41,11%

*egfr*: *epidermal growth factor recepto*r; *hh*: *hedgehog*; *wg*: *wingless*.

### Expression and function of *wg* in *Limnoporus* legs

**Figure 5:**
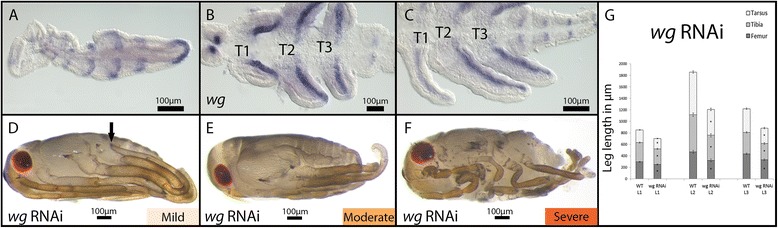
*wg* expression and function in embryonic leg development. **(A)** *wg* mRNA is expressed in early embryos in a segmental pattern along the anterior posterior axis. **(B)** In embryos with limb buds, *wg* mRNA expands to the developing limb buds and is expressed in a proximal-distal stripe, which persists through later stages **(C). (D)** Mild *wg* RNAi results in little patterning defect, but leg length is affected where T2 legs do not reach till the head (arrow). **(E)** Leg length is increasingly affected in moderate *wg* RNAi embryos. **(F)** Severely affected *wg* RNAi embryos show segmentation defect such that the boundaries between segments are no longer discernable. In addition, the legs of these embryos appear thinner and curved. **(G)** The legs of mild *wg* RNAi embryos are significantly shorter than WT. Leg measurements for T1 legs, T2 legs, and T3 legs are represented in the different bars; the three measured leg segments - the femur, the tibia, and the tarsus - are represented in the segments of each bar. *N* = 10; error bars represent ±1 standard error. An ANCOVA test was performed to compare the leg length of phenotype embryos to their WT counterparts. An asterisk indicates that the test was significant at (*P* ≤ 0.01). RNAi: gene knockdown using ribonucleic acid interference; T1, 2, 3: thoracic segments 1, 2, 3; *wg*: *wingless*; WT: wild type.

*wg* is expressed in a segmental pattern across the germband and the growth zone in early embryos before limb bud formation (Figure 5A). This segmental expression persists and extends as a stripe along the proximo-distal axis of the legs (Figure 5B,C), an expression pattern that is conserved compared to the closely related heteropteran relative *Oncopeltus* [11]. In *wg* RNAi (Figure 5D,E,F,G), the most affected embryos show defects in segmentation (Figure 5E,F). These segmentation defects were also observed in *Oncopeltus* [11] and *Tribolium* [13]. In addition, severely affected embryos showed defects in eye development reminiscent to a phenotype reported in *Oncopeltus* [11]. Mild and moderate *wg* RNAi embryos, however, showed little if any patterning defects and mainly leg length defects (Figure 5D,E). In these mildly affected embryos, all leg segments are present, but the legs are significantly shorter compared to control embryos (Figure 5D,E,G; Table 1). This shortening affects all three thoracic legs and all the segments within each of them (*P* ≤ 0.01; Table 1). This suggests that *wg* functions to promote elongation of all legs and all segments within the legs during *Limnoporus* embryogenesis.

### Expression and function of *egfr* in *Limnoporus* legs

**Figure 6:**
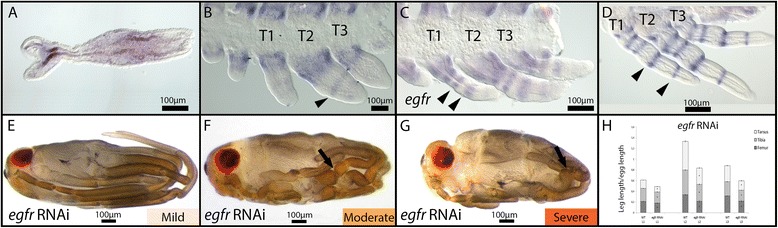
*egfr* expression and function in embryonic leg development. **(A)** *egfr* mRNA is expressed in early embryos along the germband in both head and thoracic regions. **(B)** As the limb buds appear, *egfr* mRNA can be detected in the proximal regions of the limbs, and starts to resolve into rings along the proximal-distal axis (arrowhead). **(C)** As the legs extend, the number of rings increases, until the formation of five prominent rings that prefigure the junctions between leg segments **(D). (E)** Mildly affected *egfr* RNAi embryos show little segmentation defects and only leg length appears to be affected. **(F)** Moderately and **(G)** severely affected *egfr* embryos show segmentation defects in addition to a femur specific constriction (arrow). **(H)** The legs of mild *egfr* RNAi embryos are dramatically shorter than WT. Leg measurements for T1 legs, T2 legs, and T3 legs are represented in the different bars; the three measured leg segments - the femur, the tibia, and the tarsus - are represented in the segments of each bar. *N* = 10; error bars represent ±1 standard error. An asterisk indicates that the test was significant at (*P* ≤ 0.01). *egfr*: *epidermal growth factor recepto*r; RNAi: gene knockdown using ribonucleic acid interference; T1, 2, 3: thoracic segments 1, 2, 3; WT: wild type.

The signaling receptor *egfr* is expressed in the head and the thorax in early embryos before the formation of limb buds (Figure 6A). Later on, we detected *egfr* mRNA in proximal appendages, slowly resolving in proximal-distal stripes (arrowhead in Figure 6B). As the appendages extend, *egfr* stripes become more visible and form five distinct rings along the proximal-distal axis of the three thoracic legs (Figure 6C,D). These rings seem to define the junctions between different leg segments [5]. In *egfr* RNAi (Figure 6E,F,G,H), strongly affected embryos showed severe segmentation defects and reduced appendage growth. All leg segments in these embryos are present, but their length seems severely reduced (Figure 6E,F,G; Table 1). We also observed a defect specific to the femur where a constriction forms in the middle of that segment (arrow in Figure 6F,G) in accordance with the fact that the first ring of expression in early embryos appears in proximal limb buds (Figure 6B,C). Moderately and mildly affected embryos show little or no defects in segmentation, while appendage growth defects persist (Figure 6E,F,H; Table 1). In these embryos, all leg segments are also present but are dramatically shortened (Figure 6H; Table 1). This shortening affects all three thoracic legs and all the segments within each of them (*P* ≤ 0.01; Table 1). This suggests that *egfr* promotes the growth of all legs and all segments within the legs during *Limnoporus* embryogenesis.

### Efficiency and specificity of RNAi knockdown

We confirmed the efficiency of RNAi by verifying the depletion of mRNA in the embryo for two out of the four genes studied. To do this, we stained RNAi embryos with a mix containing the probe of the gene we knocked down mixed with a tenfold diluted *egfr* probe that serves as an internal control (Figure 2). Control embryos show clear expression of *hh* mRNA (Figure 2A) and *wg* mRNA (Figure 2C) along with *egfr* mRNA as the internal control. However, both *hh* RNAi (Figure 2B) and *wg* RNAi (Figure 2D) embryos show a severe reduction in the expression of *hh* mRNA and *wg* mRNA despite the clear expression of the internal control (*egfr*). In addition, we designed double-stranded RNA from two non-overlapping fragments of * dpp*. Injection of double-stranded RNA from these two fragments induced the same phenotypes. This indicates that our RNAi procedures do not suffer from off-target effects. Altogether, our RNAi experiments indicate that, in addition to their role in segmentation, *hh*, *wg*, and *egfr* contribute to the dramatic growth of the thoracic appendages in *Limnoporus* embryos.

## Discussion

The challenging environment of the water surface has driven the evolution of a set of adaptive phenotypes that are associated with locomotion on the fluid substrate. Among these adaptations is the dramatic growth of thoracic appendages, particularly T2 and T3 legs, associated with generating efficient propulsion on the water surface (Figure 1C). Hatched nymphs are required to have immediately functional legs as they live on the water surface and employ the same mode of locomotion as adults through simultaneous rowing strokes and the generation of vortices to propel themselves on the water [14,15]. This ecology requires that the legs are specified and properly scaled during embryogenesis before hatching. In water striders, mild RNAi against *wg* and *egfr* significantly shortens thoracic appendages, while causing little leg patterning defects. *wg* RNAi affects segmentation but not appendage development in *Oncopeltus* [11] and causes the loss of thoracic appendages in *Tribolium* [13], while it failed to produce any phenotypes in the cricket [16]. Egfr patterns distal legs in flies [17] and both proximal and distal legs in *Tribolium* [18]. These observations suggest that in water striders these molecules are required for both growth and patterning the long legs.

We have also shown that during *Limnoporus* embryonic development, *hh* plays an important role in both leg segment specification and growth modulation. Hh [19,20] has a well-described function as a secreted morphogen [21] controlling patterning and segment polarity in *Drosophila* [22-24]. The expression pattern and function of *hh* is, to a great extent, conserved among insects and other arthropods [12,25,26], although this conservation has been mainly concluded by comparing *hh* expression patterns in different arthropods [25]. In *Tribolium*, *hh* functions in segmentation although there was no evidence that the segments of RNAi embryos have a polarity defect and RNAi resulted in generally smaller embryos than WT [12]. Moreover, mild *hh* RNAi in *Tribolium* had little or no effect on leg development [12]. In the cricket *Gryllus*, however, embryonic RNAi failed to deplete the *hh* transcript and to subsequently give a detectable phenotype [16]. In *Limnoporus* embryos, the expression pattern of *hh* is segmental and is distributed as stripes in the posterior compartment of each segment and along the proximo-distal axis of the thoracic legs. Analysis of *hh* RNAi phenotypes revealed a function of *hh* in segmentation, but unlike *Tribolium* [12], mildly and moderately affected embryos show a defect in both patterning of distal leg segments as well as growth of proximal segments. Our results suggest that, in addition to its role in pattern formation, *hh* contributes to the modulation of leg allometry in the embryos of water striders.

## Conclusions

The fine-tuning of the relative length of mid- and rear legs relies primarily on Ubx through its differential levels of expression between T2 and T3 legs and the sensitivity of leg tissues to Ubx protein levels [5]. Although the levels of expression of these genes change slightly in *Ubx* RNAi [5], our present data suggest that this fine-tuning of length differences between T2 and T3 legs may not be dependent on the set of developmental genes examined here. It is therefore possible that during the evolution of surface-rowing insects, other batteries of genes may have been mobilized to modulate the dramatic elongation of the mid- and rear legs. Future studies should compare the differences in gene activity between the legs of water striders and closely related terrestrial heteroptera that specialize in terrestrial habitats.

## Abbreviations

Dpp: signaling molecule decapentaplegic
Egfr: epidermal growth factor receptor
Hh: signaling molecule hedgehog
RNAi: gene knockdown using ribonucleic acid interference
Wg: signaling molecule wingless
